# Instrumented Balance Error Scoring System in Children and Adolescents—A Cross Sectional Study

**DOI:** 10.3390/diagnostics14050513

**Published:** 2024-02-28

**Authors:** Nils K. T. Schönberg, Julius Poppel, David Howell, Johanna Wagner, Michael Höfinger, Nicole Fabri, Elena M. Bonke, Philine Rojczyk, Matthias Hösl, Lorenz Kiwull, Sebastian A. Schröder, Astrid Blaschek, Katharina Vill, Inga K. Koerte, Doreen Huppert, Florian Heinen, Michaela V. Bonfert

**Affiliations:** 1 Department of Pediatric Neurology and Developmental Medicine and LMU Center for Children with Medical Complexity, Dr. von Hauner Children’s Hospital, LMU Hospital, Ludwig-Maximilians-Universität München, 80337 Munich, Germanykiwull@serious-gaming.net (L.K.);; 2 Department of Orthopedics, University of Colorado School of Medicine, Colorado Children’s Hospital, Sports Medicine Center, Aurora, CO 80045, USA; 3 cBRAIN, Department of Child and Adolescent Psychiatry, Psychosomatic and Psychotherapy, LMU Hospital, Ludwig-Maximilians-Universität München, 80337 Munich, Germany; 4 Graduate School of Systemic Neurosciences, Ludwig-Maximilians-Universität München, 80337 Munich, Germany; 5 Psychiatry Neuroimaging Laboratory, Department of Psychiatry, Brigham and Women’s Hospital, Harvard Medical School, Boston, MA 02115, USA; 6 Gait and Motion Analysis Laboratory, Schoen Clinic Vogtareuth, 83569 Vogtareuth, Germany; 7 Institute for Transition, Rehabilitation and Palliation, Paracelsus Medical University Salzburg, 5020 Salzburg, Austria; 8 Clinic for Child Neurology and Social Pediatrics, Kinderzentrum Maulbronn gGmbH, 75433 Maulbronn, Germany; 9 German Center for Vertigo and Balance Disorders, LMU Hospital, Ludwig-Maximilians-Universität München, 80337 Munich, Germany

**Keywords:** posturography, posture, postural control, body sway, center of pressure

## Abstract

**Background:** The Balance Error Scoring System (BESS) is a commonly used method for clinically evaluating balance after traumatic brain injury. The utilization of force plates, characterized by their cost-effectiveness and portability, facilitates the integration of instrumentation into the BESS protocol. Despite the enhanced precision associated with instrumented measures, there remains a need to determine the clinical significance and feasibility of such measures within pediatric cohorts. **Objective:** To report a comprehensive set of posturographic measures obtained during instrumented BESS and to examine the concurrent validity, reliability, and feasibility of instrumented BESS in the pediatric point of care setting. **Methods:** Thirty-seven participants (18 female; aged 13.32 ± 3.31 years) performed BESS while standing on a force plate to simultaneously compute stabilometric measures (instrumented BESS). Ellipse area (EA), path length (PL), and sway velocity (VM) were obtained for each of the six BESS positions and compared with the respective BESS scores. Additionally, the effects of sex and age were explored. A second BESS repetition was performed to evaluate the test–retest reliability. Feedback questionnaires were handed out after testing to evaluate the feasibility of the proposed protocol. **Results:** The BESS total score was 20.81 ± 6.28. While there was no statistically significant age or sex dependency in the BESS results, instrumented posturography demonstrated an age dependency in EA, VM, and PL. The one-leg stance on a soft surface resulted in the highest BESS score (8.38 ± 1.76), EA (218.78 cm^2^ ± 168.65), PL (4386.91 mm ± 1859.00), and VM (21.93 mm/s ± 9.29). The Spearman’s coefficient displayed moderate to high correlations between the EA (rs = 0.429–0.770, *p* = 0.001–0.009), PL (rs = 0.451–0.809, *p* = 0.001–0.006), and VM (rs = 0.451–0.809, *p* = 0.001–0.006) when compared with the BESS scores for all testing positions, except for the one-leg stance on a soft surface. The BESS total score significantly correlated during the first and second repetition (rs = 0.734, *p* ≤ 0.001), as did errors during the different testing positions (rs = 0.489–0.799, *p* ≤ 0.001–0.002), except during the two-legged stance on a soft surface. VM and PL correlated significantly in all testing positions (rs = 0.465–0.675, *p* ≤ 0.001–0.004; (rs = 0.465–0.675, *p* ≤ 0.001–0.004), as did EA for all positions except for the two-legged stance on a soft surface (rs = 0.392–0.581, *p* ≤ 0.001–0.016). A total of 92% of participants stated that the instructions for the testing procedure were very well-explained, while 78% of participants enjoyed the balance testing, and 61% of participants could not decide whether the testing was easy or hard to perform. **Conclusions:** Instrumented posturography may complement clinical assessment in investigating postural control in children and adolescents. While the BESS score only allows for the consideration of a total score approximating postural control, instrumented posturography offers several parameters representing the responsiveness and magnitude of body sway as well as a more differentiated analysis of movement trajectory. Concise instrumented posturography protocols should be developed to augment neuropediatric assessments in cases where a deficiency in postural control is suspected, potentially stemming from disruptions in the processing of visual, proprioceptive, and/or vestibular information.

## 1. Introduction

Traumatic brain injury represents a major public health issue and is a common injury during childhood and adolescence [1,2,3]. Most pediatric head injuries in high-income countries are classified as mild traumatic brain injury (mTBI), with steadily increasing numbers presenting to emergency departments [4,5].

mTBI symptoms are typically transient and resolve within days or a few weeks [6]. However, some patients complain about persisting physical, cognitive, socio-emotional, behavioral, or sleep-related symptoms [7]. About 30% of patients experience symptoms up to at least four weeks, 10% to 12 weeks, and 5% still one year after having sustained a mTBI [8,9]. To date, no biomarkers have been identified that reliably predict the course of recovery of an individual patient [10].

Balance-related symptoms (i.e., dizziness, vertigo) are commonly reported in the context of acute mTBI [11]. These symptoms may correlate with neurological findings (i.e., signs of postural instability). However, subtle impairments may be missed during a physical exam [12]. On the one hand, such minor impairments may increase the risk of falls or accidents, particularly in situations of shared attention (i.e., during sports or conversations) [13,14,15]. On the other hand, balance impairments in the acute period after mTBI have been associated with the persistence of postconcussive symptoms [16]. Therefore, sensitive point of care measures may be a reasonable addition to the routine physical examination after mTBI to assess for acute balance impairments, the trajectory of recovery, and help guide rehabilitation [17].

Regarding sports, the Balance Error Scoring System (BESS) is recommended as a sideline tool to support return to play decisions after a suspected head injury during training or competition [18,19,20,21,22]. As BESS is an observer-rated clinical tool susceptible to rating bias, more advanced tools for assessing postural stability may be more appropriate and sensitive in the clinical context [23,24]. In adult neurology and sports medicine, the instrumented assessment by force plates is established [11,25,26,27,28,29]. The BESS combined with an instrumented posturographic assessment may also offer a more comprehensive evaluation of postural stability in pediatric patients. However, to date, no standardized protocols for instrumented posturography in the pediatric point of care setting are available, even though an association of concussion-like symptoms and instrumented balance performance in a large sample of athletes aged 9 to 18 years has been previously reported [30]. 

Here, we present the first-ever data of a cross-sectional clinical study designed to explore the validity, test–retest reliability, and feasibility from the children’s perspective of an instrumented assessment of postural control in children and adolescents: the instrumented BESS. For the instrumented BESS, the participants performed the six different BESS standing positions on a force plate while posturographic measures as a surrogate of body sway were collected. Besides the BESS total scores, the variability of the body sway during the different testing positions as well as sex and age-dependent differences in postural performance were analyzed. This study not only adds comparative data to the currently scarce body of literature regarding posturography in children and adolescents, but also provides insights into reasonable adoptions for different groups of patients.

## 2. Materials and Methods

### 2.1. Ethical Approval

Ethical approval was obtained from the internal review board (vote 20–160). Informed written consent of the participants and legal guardians were a prerequisite for study inclusion. 

### 2.2. Participants

Children and adolescents who had presented to a tertiary pediatric center due to mTBI (defined in line with the WHO mTBI task force) were screened to participate in the study [31]. Inclusion criteria were age of 6 to 17 years, time since mTBI > 12 weeks, non-protracted recovery (<4 weeks) and complete clinical recovery (Kings Outcome Scale of Head Injury 5b; no more complaints and symptoms related to the mTBI), and no history of any neurological, neurodevelopmental, neuropsychiatric disorders, or chronic internal disease [32].

### 2.3. BESS

Before testing, the participants were instructed to execute a ball-kicking task for the determination of their dominant foot. All assessments were conducted without footwear. Six distinct testing positions were evaluated in a specified sequence, encompassing three standing tasks (bipedal with feet hip-width apart, unipedal on the non-dominant foot, and tandem stance with the non-dominant foot positioned at the rear) each lasting 20 s. Each test was executed under two conditions—on both a firm and soft surface (foam pad (48 cm × 40 cm × 6 cm, density: 38.6 kg/m^3^, AIREX^®^, Sins, Switzerland)) with closed eyes. Children were instructed to interrupt testing in the event of discomfort or fatigue. Recorded errors, documented on paper, included hands lifting off the iliac crests, opening of the eyes, taking a step, stumbling, or falling, moving the hip beyond 30 degrees of abduction or flexion, lifting the forefoot or heel, or deviating from the test position for more than 5 s. A predefined maximum of 10 errors was established for each trial, resulting in a maximum possible error count of 60 for the overall BESS score across all six testing positions. All evaluations were conducted by two examiners who had received training in the instrumented BESS testing protocol. 

### 2.4. Posturography

**Figure 1:**
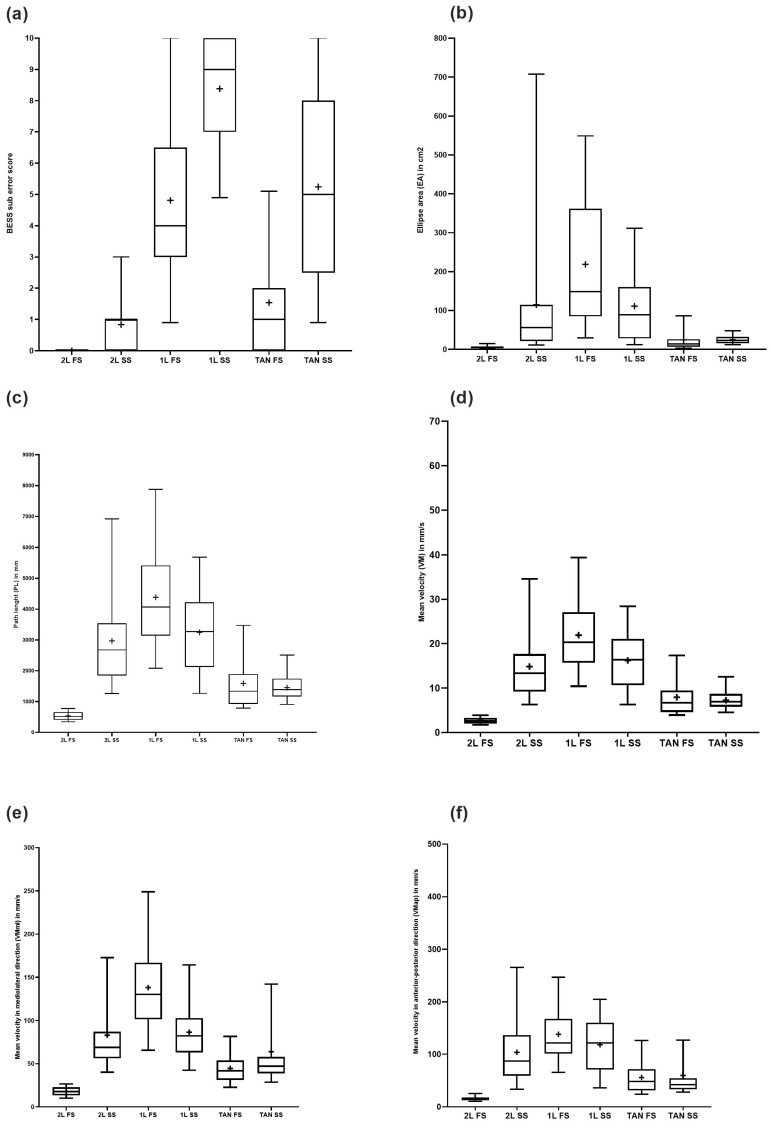
Box and whisker plots of (**a**) the BESS scores and posturographic parameters, (**b**) ellipse area in cm^2^, (**c**) path length in mm, (**d**) mean velocity in cm/s, (**e**) mean velocity in mediolateral direction in cm/s, and (**f**) mean velocity in anterior–posterior direction in cm/s) in all six testing positions. Testing positions: two-legged stance on a firm surface (2L FS), one-legged stance on firm surface (1L FS), tandem stance on firm surface (Tan FS), two-legged stance on a soft surface (2L SS), one-legged stance on a soft surface (1L SS), tandem stance on a soft surface (Tan SS). All graphs are shown with the firm and soft surface values side by side for each testing position to allow for a better comparison. Lower and upper error lines show the 5th and 95th percentiles, respectively; means are represented by +.

During the BESS, postural stability was quantified by instrumented posturography (Leonardo Mechanograph^®^ GRFP LT force plate, Novotec Medical GmbH, Pforzheim, Germany) (Figure 1). Data were sampled at 500 Hz and analyzed by BAS Edition V4.4b03.42 software (BAS Version V4.4b03.42, Novotec Medical GmbH, Pforzheim, Germany).

Prior to testing, the force plate was calibrated by utilizing the integrated calibration-application and was leveled to account for potential uneven ground. Manual taring of the force plate setup was performed to compensate for weight variations among the participants, both initially and following the incorporation of the foam pad into the assessment. Utilizing the force distribution data from its four sensors, the effective center of pressure (CoP) was computed for each sample point. Leonardo mechanography reduces the measurement data to 100 Hz before conducting the CoP analysis [33]. Outcome parameters derived from the CoP as an approximation of the participant’s ability to stabilize their center of mass [34] were as follows: area of the standard ellipse (EA) accounting for 90% of all CoP points during body excursion in cm^2^, CoP path length in mm (PL) as well as the CoP mean sway velocity in mm/sec (VM). The latter was more specifically explored in the anterior–posterior (Vmap) and mediolateral (VMml) directions. The chosen parameters represented the spatial variability of balance data on a two-dimensional surface and are surrogates for the quantitative magnitude (EA) and responsiveness (VM) of body sway as well as for the movement trajectory (PL). This method of analysis proved user-friendly due to its easy interpretation in previous studies [35].

### 2.5. Feedback 

At the end of the testing session, the participants completed paper-based feedback forms to determine whether the testing procedure was user-friendly from the participants’ perspective. The questionnaire included nine Likert-scaled questions (rated on a scale of 1 to 5) pertaining to the testing procedure. An open comment section was incorporated to allow for suggestions aimed at enhancing the testing process. For younger children, assistance in completing the form was provided by their caregiver.

### 2.6. Statistical Analysis

Statistical analyses were performed with Statistical Package for the Social Sciences (SPSS version 28; SPSS Inc., Chicago, IL, USA). Normally distributed data are presented as the mean and standard deviation; not normally distributed data are presented as the median and interquartile range. The level of significance was set to 0.05. Outliers of posturographic measures were eliminated if a threshold of three standard deviations was violated (exclusion of the dataset of one participant due to incompliance/distraction). Repeated-measures ANOVA together with Bonferroni-adjusted post hoc analysis was used to test for differences of BESS and posturographic measures among positions. Differences of Vmap and VMml were examined by the paired t-test. Posturographic cumulative total scores for EA, VM, and PL were each computed by summing up the respective data of all six testing positions. Participants were categorized by sex and age (<12 years or ≥12 years) and differences assessed by appropriate two group testing. Concurrent validity of EA, VM, and PL with non-interval error counts of BESS was analyzed by Spearman’s rank correlation coefficient (rs: very weak <0.19; weak 0.20–0.39; moderate 0.40–0.59; strong 0.60–0.79; very strong 0.80–1.0) [36]. Test–retest reliability of the instrumented BESS was analyzed using Spearman’s rank-correlation, effect sizes were interpreted according to Cohen with an effect size of r < 0.10 indicating a small effect, r < 0.30 indicating a medium effect, and r < 0.50 indicating a large effect [37]. Feedback questionnaires were analyzed by descriptive statistics.

## 3. Results

### 3.1. Study Population

Thirty-seven participants (18 females) aged 13.32 ± 3.31 years (n = 11 < 12 years) who had completely recovered from non-protracted mTBI (time since injury 5.35 ± 2.92 years (range 3–123 months)) performed the instrumented BESS. 

### 3.2. BESS

**Figure 2:**
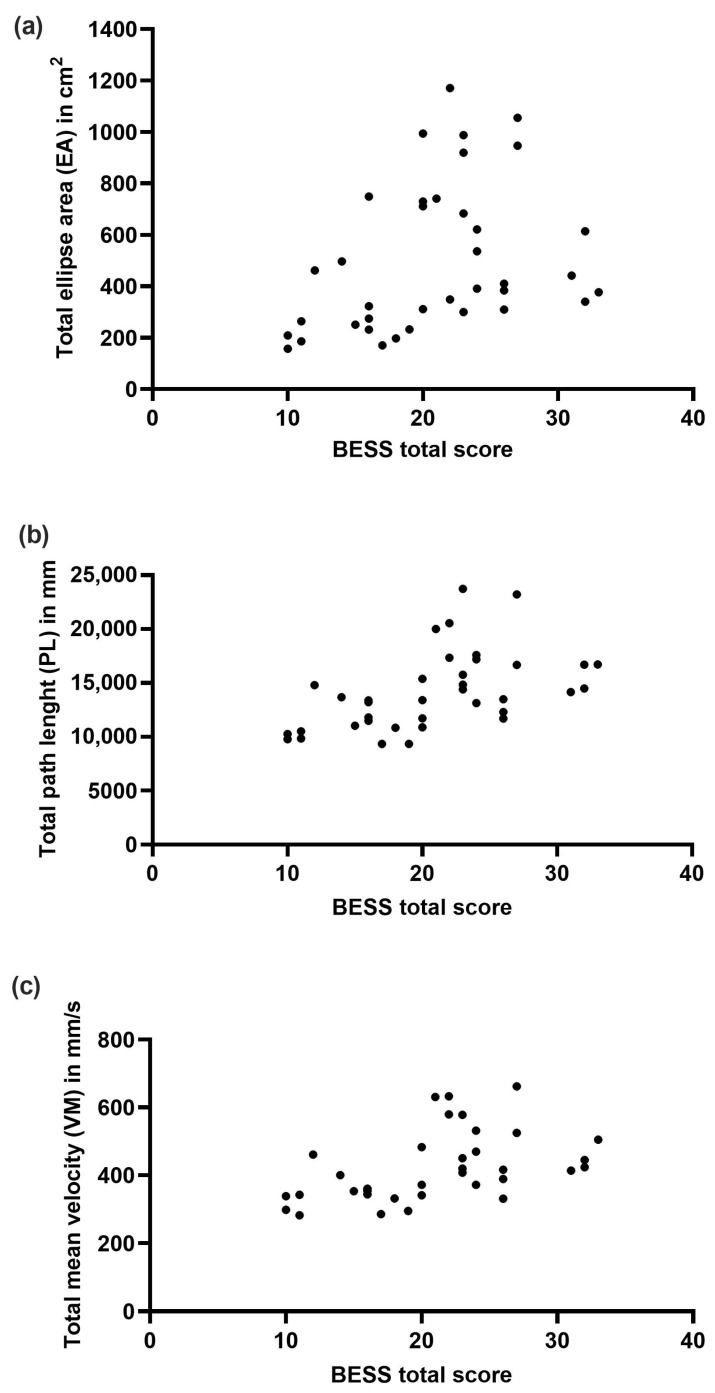
Scatter plots of the correlation of the BESS total score and cumulative total posturographic measures. BESS total scores and individually calculated cumulative total posturographic scores being the sum of all testing positions for each parameter for each patient were correlated and graphed via a X-Y-Table. Cumulative total parameter scores were calculated for (**a**) ellipse area in cm^2^, (**b**) path length in mm, and (**c**) mean velocity in cm/s.

The BESS total score yielded 20.81 ± 6.28 (Table 1). Most errors were recorded in a one-legged stance a on soft surface (8.38 ± 1.76), with 45.9% of all participants experiencing a maximum error score of 10 in this condition (Table 1 and Figure 2). During the one-legged stance on a firm surface, 18.9% of participants recorded the maximum errors, and this was the case in 10.8% of tandem stance trials. No participant received the maximum 10 errors during the two-legged stance on a soft and firm surface, or during the tandem stance on a firm surface. Across all test positions, 40.3% of all errors occurred during the one-legged stance on a soft surface. Repeated measures ANOVA revealed a significant difference in BESS errors (*p* = 0.002; Supplementary Table S1). Overall, each testing position differed from all others, except for the one-legged stance on a firm surface not differing from a tandem stance on a soft surface, and a tandem stance on a firm surface not differing from a two-legged stance on a soft surface (Supplementary Table S2). The BESS scores did not significantly differ between the sex: and age groups (Table 1). 

### 3.3. Posturography

The highest body sway was recorded in the one-legged stance on a soft surface, followed by the one-legged stance on a firm surface and tandem stance on a soft surface (Table 2, Figure 1). 

Repeated measures ANOVA resulted in significant differences for all posturographic measures within the different testing positions (*p* = 0.001; Supplementary Table S1). In line with the BESS scores, no significant difference was detected for any of the measures between the one-legged stance on a firm surface and the tandem stance on a soft surface, and between the tandem stance on a firm surface and the two-legged stance on a soft surface (Supplementary Tables S3–S7). In addition, the PL and VM did not significantly differ between the one-legged stance on a soft surface and tandem stance on a soft surface. Regarding Vmap, no difference was detected between the one-legged stance on a firm surface and two-legged stance on a soft surface as well as for the one-legged stance on a soft surface and tandem stance on a soft surface. VMml did not differ between the one-legged stance on a firm surface and two-legged stance on a soft surface as well as the two-legged stance on a soft surface and tandem stance on a soft surface. 

Younger participants demonstrated significantly higher body sway and velocity of CoP change than adolescents regarding some parameters and testing conditions (Table 2). No differences between the male and female participants were demonstrated for the EA, VM, and PL in any testing position (Table 2). Additionally, VM was differentially analyzed for the anterior–posterior and mediolateral components (Table 3). Comparisons of Vmap and VMml presented significant differences in all testing positions except the one-legged stance on a soft surface with the VM being higher in the mediolateral direction for the two-legged stance on a firm (*p* < 0.001) and soft surface (*p* = 0.004). Sway velocity was higher in the anterior–posterior direction during the one-legged stance on a firm surface (*p* < 0.001), one-legged stance on a soft surface (*p* < 0.001), and tandem stance on a soft surface (*p* < 0.001; Supplementary Table S8). Age group differences were observed, with participants younger than 12 years of age delivering higher VMml in the one-legged stance on a firm (*p* = 0.014) and soft surface (*p* = 0.002); Vmap in the two-legged stance on a firm (*p* = 0.031) and soft surface (*p* = 0.012) as well as the tandem stance on a firm surface (*p* = 0.017). Regarding sex, group differences were only detected in VMml in the tandem stance on a firm surface with higher velocities in boys than girls (*p* = 0.019; Table 3). 

Finally, the posturographic total score was significantly different regarding the EA, VM, and PL between the two age groups (*p* = 0.008–0.014), with the younger age group yielding higher total scores. No significant differences between the male and female participants were observed (Table 2). 

### 3.4. Correlation of BESS and Force Plate Measures

Spearman’s rank coefficients demonstrated moderate to very strong correlations between the EA, PL, and VM and BESS errors in all testing positions but the one-legged stance on a soft surface (weak correlation, Table 4). The two-legged stance on a firm surface was not considered due to the mean of zero error scores in this BESS position. Cumulative total posturographic scores for the EA, VM, and PL all demonstrated a positive moderate to strong correlation with the BESS total scores (r_s_ = 0.46–0.60, *p* < 0.004, Figure 2).

### 3.5. Test–Retest Reliability of Instrumented BESS

The BESS total score correlated significantly during the first and second repetition (rs = 0.734, *p* ≤ 0.001), corresponding to a large effect. Errors during the different BESS testing positions correlated significantly in repetition one and two, except during the two-legged stance on a soft surface (rs = 0.489–0.799, *p* ≤ 0.001–0.002), corresponding to a medium to large effect. The correlation for the two-legged stance on a firm surface was not calculable due to no occurrence of errors. EA in repetition one and two correlated significantly in all testing positions, except for the two-legged stance on a soft surface (rs = 0.392–0.581, *p* ≤ 0.001–0.016), corresponding to a medium to large effect. VM in repetition one and two correlated significantly in all testing positions (rs = 0.465–0.675, *p* ≤ 0.001–0.004), corresponding to a medium to large effect. PL in repetition one and two correlated in all testing positions (rs = 0.465–0.675, *p* ≤ 0.001–0.004), corresponding to a medium to large effect (Table 5).

### 3.6. Feedback

The majority of all participants (92%) reported that the instructions for the balance testing procedure were very well-explained before testing commenced. However, testing on the force plate left a significant portion (61%) of participants undecided on whether the balance testing procedure was easy for them. A total of 78% of participants reported enjoying the balance testing experience at our facility. The balance testing reminded most of the children of either a medical appointment or a physical education lesson (Table 6).

## 4. Discussion

This study is the first to comprehensively assess balance performance by instrumented BESS in children and adolescents. So far, reports regarding the validity and reliability of this approach have only been limited to one posturographic parameter in all testing positions: the mean sway velocity (VM) of the center of pressure (CoP) [38]. In the current study, we expected an increasing postural sway corresponding to the increasing difficulty levels of the six BESS standing positions (firm < foam surface; bipedal < tandem < single leg stance), reflecting a higher need for adjustments of the center of pressure over the base of support. In addition to these analyses regarding numerical differences in body sway, we investigated the concurrent validity between the BESS outcomes and the simultaneously recorded instrumented posturographic measures to establish their correlation. Furthermore, the test–retest reliability of the instrumented BESS was evaluated. Last but not least, the participants were asked to provide feedback to assess for the need of further age-appropriate adoptions of instructions and testing procedures. 

### 4.1. BESS 

The average BESS total score was in alignment with the results of previous pediatric studies [39,40,41]. Of note, neither the BESS pass or fail cut-off nor age-dependent reference data have been established. While some authors have reported an age-related decrease in BESS scores, other groups and this study did not demonstrate such age-dependency [39,40,41]. These contradicting findings may be explained by the variance in age ranges of the participants in the different studies. The available data indicate that the younger the population, the higher the variance of performance in postural control, depending on the level of maturation of the sensorimotor system [42]. Some previous studies have demonstrated sex-dependent differences in BESS scores [43] while others including this study did not replicate this finding [44,45]. 

### 4.2. Posturography

The current study reports the EA, VM, Vmap, VMml, and PL in all the BESS testing positions together with a total posturographic score for each of the measures to allow for a differentiated analysis of magnitude (expressed as EA), responsiveness of body sway (expressed as VM), and movement trajectory (expressed as PL). Like the BESS scores, instrumentally recorded body sway significantly increased with the level of demand of the test conditions. VM as well as VM in AP and ML in the two-legged stance, both on a soft and firm surface, were comparable to data reported by other researchers [42,46,47]. Compared to percentiles for young adults, our cohort yielded higher PL, likely reflecting not yet completely matured postural mechanisms in our group of children and adolescents [48]. In contrast to previous reports, females did not perform better than males in this study, except for VMml in the tandem stance on a firm surface [43,45,49]. In line with previous studies, the parameters of postural control tended to improve with age, translating to statistically significant differences in several testing positions between age groups [50,51]. Overall, compared to the BESS, instrumented posturography proposes a higher discriminatory power regarding age [52].

VMml has been reported to yield higher results than VMap, particularly in younger participants [53]. This finding was replicated in this study for the double stance position on a soft and firm surface. However, for the one-legged stance on a soft surface and tandem stance on a firm and soft surface, the VMap exceeded VMml. While participants compensated loss of equilibrium during the two-legged stance in the mediolateral direction, loss of equilibrium in more complex testing positions was compensated by anterior–posterior sway. This finding is in line with research suggesting an earlier maturation of postural control in an anterior–posterior axis (ankle strategy) than mediolaterally (hip strategy) [53,54,55].

Next to our report, only a few small-scale studies exist that investigated more complex stance positions such as the one-legged or tandem stance in a pediatric cohort [56,57]. As expected, postural sway increases with higher demands. The use of a variety of test positions including the alteration of visual and sensory input allows for a more sensitive assessment of postural sway in the context of physiological trajectory during development as well as of neurological disorders [42,51,58,59]. Of particular interest are developmental mechanisms as well as disorder-related alterations of the processes of integration of visual, proprioceptive, and vestibular information for maintaining postural control. Sensory inputs as well as neuromuscular feedback loops are steadily reweighted, but the extent to which the single sense is considered differs during motor maturation [60,61]. Further research in different sensory conditions for different age groups is warranted to better classify the respective performance on the force plate. 

Given the novelty of pediatric posturographic studies, up to now, no consensus has been achieved regarding the clinical relevance and the specific selection of the measures to report or several technical aspects (e.g., time spent per test position or sampling rate). The design of the balance assessment and the selection of posturographic measures to analyze in this study were based on a thorough literature review and exchange with experts in the field as we are aiming at introducing such an instrumented balance assessment in our pediatric concussion care pathway. However, research in the field of instrumented posturography is quite heterogenous, as highlighted by the work of Pilz et al. They only very recently published normative reference data for children and adolescents between 4 and 17 years of age for the following test positions: Romberg test eyes open, Romberg test eyes closed, semi-tandem test eyes open, semi-tandem test eyes closed, tandem test eyes open, tandem test eyes closed, and one-leg stand test eyes open for 10 s each. Based on their data, Pilz et al. identified six outcome parameters deemed suitable to quantify static postural performance: VM as a parameter of the velocity histogram (vCoFmean), the equilibrium score (ML and AP), and the sway angle SD (ML and AP) [57]. Other research groups have focused their analysis on the sway index [30]. Against this background, future investigations are highly needed to explore the significance of the numerous available posturographic measures and derived parameters in different pediatric populations. For sure, the establishment of normative percentiles would be invaluably helpful to categorize the performance of one single patient at a single timepoint as normal or impaired.

### 4.3. Correlation of BESS and Force Plate Measures

Moderate to good correlations between all three posturographic variables (EA, VM, PL) and the BESS scores were demonstrated in this study, supporting the concurrent validity of the instrumented posturography using a force plate system with clinically observer-rated errors during BESS. This is in line with a previous study demonstrating the correlation of BESS and VM on a portable force plate [38,62]. No previous reports exist for EA and PL in which to compare our findings [51].

The strength of correlation between the BESS scores and magnitude of body sway differed depending on the testing position. On the one hand, basement effects are likely to play a role. On the other hand, in tests that are easy to perform, the likelihood of BESS errors is lower despite potential difficulties in postural stabilization. Body sway is more easily compensated for in less demanding conditions (i.e., two-legged or tandem stance on a firm surface) with minor compensating movements. Even though these movements may not be detected by observer-rated tools and do not qualify as BESS errors, they may play a role in the performance of everyday and sports physical activities and are detectable via instrumented posturography.

Interestingly, posturographic measures collected during the one-legged stance on a soft surface, which highly challenges participants, demonstrated no statistically significant correlation between the BESS scores and EA, VM, or PL. This may be explained by the total BESS error scores being limited to a maximum of 10, while posturographic measures allow for a more sensitive and precise measurement of body sway without any limitation. BESS limitations in patients who have inadequate postural control—thus a high BESS score—can be overcome by combining it with instrumented posturography. This combination enables a very careful second look at the distinct degree and kind of imbalance. Additionally, BESS is susceptible to learning and tiring effects [23,63]. While instrumented posturography is also predisposed to learning effects, it is not prone to ceiling effects due to the open-ended nature of its scale. Furthermore, while BESS error scoring is not age-adopted, posturographic results could be interpreted in the context of age specific percentiles as soon as they become available, allowing for a very precise analysis of postural capability. The BESS has demonstrated a good interrater reliability, but previous studies have questioned its objectivity [64]. Given this background, short protocols of tailored instrumented posturographic testing have the potential to add relevant information to the clinical examination of children and adolescents with different neurological disorders in different clinical settings.

### 4.4. Test–Retest Reliability

The exploration of the test–retest reliability of the BESS between the first and second repetition revealed a strong correlation for the BESS total score and individual BESS errors as well as for the posturographic measures in all but one testing position. This observation is comparable to previous investigations [38,65]. This indicates that the instrumentation of the BESS is a reliable measure of postural control.

### 4.5. Feedback

Although the majority of participants felt well-prepared for the testing procedure, a considerable number of participants perceived the BESS testing as not easy to perform. This finding highlights the need for an age-adapted and standardized explanation. In addition, the introduction of a dummy test to go through the testing procedure once under “real world” conditions with the possibility to ask questions in case of uncertainty could be helpful.

### 4.6. Limitations

This cross-sectional study investigated pediatric participants with a history of mTBI with non-protracted, complete clinical recovery. Given the wide range of time that passed since mTBI together with the cross-sectional design, it was not the aim of this study to explore the trajectory of performance in postural control after mTBI. This topic should be addressed in future prospective studies exploring postural control at different time points directly and in the sequel after acute mTBI. For similar cross-sectional studies in healthy child or youth athletes, an incidental history of a previous concussion with complete clinically recovery had not affected the balance performance [30]. As there is no age-specific adoption of the testing procedure, the reliability of BESS assessment in younger children is a subject of discussion. This similarly pertains to force plate measurements, as the differentiation between pathological imbalances and the ongoing development of postural control in children is complex and requires further research. The relatively small sample size does not allow for a generalization of the findings of this study. Future studies should also include an assessment of athletic activity and body mass index to explore the interaction effects [52]. 

## 5. Conclusions

This cross-sectional study identified the instrumented BESS as a reliable and valid diagnostic tool in a point of care setting and provides insights into possibly helpful age-appropriate adoptions in explanation and procedure of testing. By combining the clinical BESS testing to instrumented posturography, this work adds valuable information regarding the magnitude, responsiveness, and trajectory of body sway in several testing positions. Short and tailored protocols of instrumented posturography should be established to complement the neuropediatric examination if an impairment of postural control due to alterations in the processing of visual, proprioceptive, and/or vestibular information is suspected. Regarding the clinical research of motor development during childhood and adolescence, instrumented posturography could also add valuable information to elucidate the maturation of sensory-motor integration and neuromuscular feedback mechanisms.

## Figures and Tables

**Table 1:** BESS subscores and total scores.

BESS Subscores and Total Scores									
Total Cohort				Group Comparison Age			Group Comparison Sex		
n = 37				<12 y.o.a.	≥12 y.o.a.		Female	Male	
				n = 11	n = 26		n = 18	n = 19	
Testing Position	Mean ± SD	95% CI	(Range)	Mean ± SD	Mean ± SD	*p*	Mean ± SD	Mean ± SD	*p*
2L FS	0 ± 0	[0; 0]	(0–0)	0	0	n.a.	0	0	n.a.
1L FS	4.80 ± 2.83	[3.87; 5.75]	(0–10)	6.36 ± 3.33	4.15 ± 2.36	0.087	4.50 ± 2.28	5.11 ± 3.30	0.660
Tan FS	1.54 ± 1.64	[0.99; 2.09]	(0–6)	2.18 ± 1.62	1.27 ± 1.59	0.065	1.33 ± 1.72	1.74 ± 1.60	0.298
2L SS	0.84 ± 1.01	[0.50; 1.18]	(0–3)	1.09 ± 1.30	0.73 ± 0.87	0.635	0.72 ± 0.75	0.95 ± 1.22	0.988
1L SS	8.38 ± 1.76	[7.79; 8.96]	(4–10)	9.18 ± 1.25	8.04 ± 1.84	0.075	8.17 ± 1.65	8.58 ± 1.87	0.327
Tan SS	5.24 ± 2.82	[4.29; 6.20]	(0–10)	4.55 ± 2.84	5.54 ± 2.87	0.342	5.28 ± 3.30	5.21 ± 2.46	0.940
BESS Total score	20.81 ± 6.28	[18.72; 22.90]	(10–33)	23.36 ± 6.01	19.73 ± 6.15	0.108	20.00 ± 5.88	21.58 ± 6.69	0.45

Years of age (y.o.a), not applicable (n.a.), two-legged stance on a firm surface (2L FS), one-legged stance on a firm surface (1L FS), tandem stance on a firm surface (Tan FS), two-legged stance on a soft surface (2L SS), one-legged stance on a soft surface (1L SS), tandem stance on a soft surface (Tan SS).

**Table 2:** Posturographic measures retrieved during instrumented BESS.

	Total Cohort	Group Comparison Age			Group Comparison Sex		
	n = 37	<12 y.o.a.	≥12 y.o.a.		Female	Male	
		n = 11	n = 26		n = 18	n = 19	
Testing Position	Mean ± SD	Mean ± SD	Mean ± SD	*p*	Mean ± SD	Mean ± SD	*p*
Ellipse Area in cm^2^							
2L FS	5.62 ± 3.35	6.55 ± 3.77	5.23 ± 3.15	0.242	5.91 ± 4.23	5.35 ± 2.33	0.869
1L FS	115.10 ± 171.0	**237.33 ± 270.12**	**63.39 ± 59.84**	**0.023**	52.39 ± 32.31	174.51 ± 223.29	0.221
Tan FS	24.7 ± 31.49	**32.57 ± 24.83**	**21.38 ± 33.80**	**0.043**	18.39 ± 18.49	30.68 ± 39.78	0.142
2L SS	25.03 ± 11.50	24.01 ± 11.07	25.49 ± 11.86	0.658	25.67 ± 13.07	24.44 ± 10.11	0.893
1L SS	218.78 ± 168.65	**313.72 ± 149.51**	**178.61 ± 162.40**	**0.007**	226.26 ± 180.86	211.69 ± 160.90	0.893
Tan SS	111.25 ± 99.30	79.07 ± 76.80	124.87 ± 105.79	0.150	126.90 ± 121.97	96.42 ± 72.06	0.775
Total Score	**500.49 ± 286.38**	**693.25 ± 317.99**	**418.94 ± 233.21**	**0.014**	455.51 ± 233.08	543.10 ± 329.82	0.425
Path Length PL in mm							
2L FS	534.11 ± 138.20	597.92 ± 141.46	507.11 ± 130.19	0.067	516.33 ± 145.70	550.96 ± 132.44	0.450
1L FS	2972.24 ± 1586.65	3884.95 ± 2235.32	2586.00 ± 1051.13	0.100	2488.67 ± 810.37	3430.36 ± 1989.80	0.220
Tan FS	1589.48 ± 773.37	**1997.36 ± 870.53**	**1416.90 ± 673.77**	**0.028**	1418.72 ± 802.35	1751.23 ± 729.00	0.066
2L SS	1454.29 ± 410.34	1485.20 ± 560.13	1441.21 ± 341.13	0.780	1413.76 ± 344.54	1392.69 ± 470.58	0.869
1L SS	4386.91 ± 1859.00	**5350.72 ± 1361.22**	**3979.15 ± 1911.45**	**0.002**	3797.31 ± 972.92	4945.48 ± 2311.38	0.210
Tan SS	3246.75 ± 1319.72	3140.53 ± 1416.29	3291.69 ± 1303.30	0.765	3216.22 ± 1470.38	3275.68 ± 1199.85	0.890
Total Score	**14,183.78 ± 3654.10**	**16,456.69 ± 3527.04**	**13,222.17 ± 3319.14**	**0.008**	12,851.01 ± 2229.90	15,446.41 ± 4306.92	0.057
Mean Velocity in mm/s							
2L FS	2.65 ± 0.70	**2.99 ± 0.70**	**2.51 ± 0.66**	**0.047**	2.58 ± 0.73	2.72 ± 0.68	0.578
1L FS	14.86 ± 7.39	19.42 ± 11.18	12.93 ± 5.26	0.100	12.44 ± 4.05	17.15 ± 9.95	0.220
Tan FS	7.95 ± 3.87	**9.99 ± 4.35**	**7.08 ± 13.37**	**0.028**	7.09 ± 4.01	8.76 ± 3.64	0.066
2L SS	7.27 ± 2.05	7.43 ± 2.80	7.01 ± 1.71	0.781	7.07 ± 1.72	7.46 ± 2.35	0.869
1L SS	21.93 ± 9.29	**26.75 ± 6.81**	**18.89 ± 9.56**	**0.002**	18.98 ± 4.86	24.72 ± 11.56	0.210
Tan SS	16.23 ± 6.60	15.70 ± 7.08	16.45 ± 6.52	0.755	16.08 ± 7.35	16.38 ± 6.00	0.893
Total Score	**434.06 ± 126.00**	**494.88 ± 106.92**	**408.33 ± 126.38**	**0.011**	406.33 ± 130.33	460.33 ± 119.21	0.066

Years of age (y.o.a), two-legged stance on a firm surface (2L FS), one-legged stance on a firm surface (1L FS), tandem stance on a firm surface (Tan FS), two-legged stance on a soft surface (2L SS), one-legged stance on a soft surface (1L SS), tandem stance on a soft surface (Tan SS). Statistically differing results are printed in bold.

**Table 3:** VMml und Vmapl retrieved during the instrumented BESS.

	Total Cohort	Group Comparison Age			Group Comparison Sex		
	n = 37	< 12 y.o.a.	≥ 12 y.o.a.		Female	Male	
		n = 11	n = 26		n = 18	n = 19	
Testing Position	Mean ± SD	Mean ± SD	Mean ± SD	*p*	Mean ± SD	Mean ± SD	*p*
Mean Velocity ML in mm/s							
2L FS	18.05 ± 5.20	19.69 ± 4.44	17.35 ± 5.42	0.216	17.87 ± 5.61	18.22 ± 4.93	0.845
1L FS	82.99 ± 44.60	**114.18 ± 65.00**	**69.79 ± 23.76**	**0.014**	66.24 ± 15.89	98.85 ± 56.52	0.070
Tan FS	44.49 ± 17.37	52.39 ± 16.73	41.14 ± 16.83	0.051	**38.34 ± 15.86**	**50.31 ± 17.09**	**0.019**
2L SS	63.80 ± 92.85	49.65 ± 21.05	69.79 ± 110.05	0.349	77.84 ± 132.43	50.50 ± 17.16	0.999
1L SS	138.29 ± 54.83	**173.69 ± 41.69**	**123.31 ± 53.38**	**0.002**	120.79 ± 30.89	154.87 ± 67.20	0.178
Tan SS	86.45 ± 33.30	85.28 ± 36.26	86.94 ± 32.71	0.892	85.24 ± 34.43	87.59 ± 33.09	0.834
Mean Velocity AP in mm/s							
2L FS	15.56 ± 4.37	**18.33 ± 4.88**	**14.38 ± 3.63**	**0.031**	14.77 ± 4.52	16.31 ± 4.20	0.159
1L FS	83.21 ± 61.13	133.38 ± 81.74	90.99 ± 46.41	0.140	89.22 ± 36.06	117.22 ± 76.44	0.480
Tan FS	55.96 ± 31.52	**74.07 ± 38.05**	**48.30 ± 25.42**	**0.017**	51.64 ± 34.62	60.04 ± 28.61	0.118
2L SS	59.86 ± 94.01	44.41 ± 14.85	66.40 ± 111.82	0.730	76.50 ± 134.06	44.07 ± 13.51	0.620
1L SS	138.17 ± 67.51	**162.95 ± 45.82**	**127.68 ± 73.06**	**0.012**	118.40 ± 35.25	156.90 ± 84.74	0.233
Tan SS	118.23 ± 53.88	112.41 ± 56.42	120.69 ± 53.71	0.675	86.84 ± 61.90	118.81 ± 46.75	0.948

Years of age (y.o.a), mean velocity (VM), mean velocity in mediolateral (VMml), mean velocity in anterior–posterior (Vmap), two-legged stance on a firm surface (2L FS), one-legged stance on a firm surface (1L FS), tandem stance on a firm surface (Tan FS), two-legged stance on a soft surface (2L SS), one-legged stance on a soft surface (1L SS), tandem stance on a soft surface (Tan SS). Statistically differing results are printed in bold.

**Table 4:** Correlation of BESS scores and posturographic measures by Spearman’s rank coefficients.

BESS Score	Ellipse Area (cm^2^)		Mean Velocity (mm/s)		Path Length (mm)	
Testing position	Spearman’s Rho (r_s_)	*p*	Spearman’s Rho (r_s_)	*p*	Spearman’s Rho (r_s_)	*p*
2L FS	%	%	%	%	%	%
1L FS	**0.61**	**<0.001**	**0.59**	**<0.001**	**0.59**	**<0.001**
Tan FS	**0.77**	**<0.001**	**0.74**	**<0.001**	**0.74**	**<0.001**
2L SS	**0.43**	**<0.001**	**0.45**	**0.005**	**0.45**	**0.005**
1L SS	0.30	0.070	0.25	0.141	0.25	0.141
Tan SS	**0.76**	**<0.001**	**0.81**	**<0.001**	**0.81**	**<0.001**
Total Score	**0.46**	**0.004**	**0.54**	**<0.001**	**0.60**	**<0.001**

% Unable to be computed due to the lack of variability in the balance error scores. Two-legged stance on a firm surface (2L FS), one-legged stance on a firm surface (1L FS), tandem stance on a firm surface (Tan FS), two-legged stance on a soft surface (2L SS), one-legged stance on a soft surface (1L SS), tandem stance on a soft surface (Tan SS). Statistically significant results are printed in bold.

**Table 5:** Instrumented BESS test–retest reliability.

BESS	First Round	Second Round	Spearman’s Rho	
	Mean ± SD	Mean ± SD	r_s_	*p*
FS 2L	0.0 ± 0.0	0.03 ± 0.167	n.c.	
**FS 1L**	**4.67 ± 2.77**	**3.81 ± 2.94**	**0.799**	**<0.001**
**FS Tan**	**1.56 ± 1.66**	**1.58 ± 1.96**	**0.798**	**<0.001**
SS 2L	0.78 ± 0.96	0.28 ± 0.62	0.249	0.143
**SS 1L**	**8.33 ± 1.76**	**8.33 ± 1.94**	**0.489**	**0.002**
**SS Tan**	**5.33 ± 2.85**	**5.03 ± 3.03**	**0.678**	**<0.001**
**Total Score**	**20.81 ± 6.28**	**18.81 ± 6.79**	**0.734**	**<0.001**
**EA**	**First Round**	**Second Round**	**Spearman’s Rho**	
	Mean ± SD	Mean ± SD	**r_s_**	***p***
**FS 2L**	**5.56 ± 3.38**	**6.04 ± 5.23**	**0.508**	**0.001**
**FS 1L**	**116.22 ± 173.32**	**104.18 ± 178.10**	**0.581**	**<0.001**
**FS Tan**	**20.68 ± 20.13**	**20.34 ± 23.05**	**0.392**	**0.016**
SS 2L	25.28 ± 11.56	17.72 ± 5.56	0.178	0.299
**SS 1L**	**221.97 ± 169.91**	**159.68 ± 142.41**	**0.435**	**0.008**
**SS Tan**	**114.04 ± 99.22**	**81.88 ± 84.98**	**0.415**	**0.012**
**PL**	**First Round**	**Second Round**	**Spearman’s Rho**	
	Mean ± SD	Mean ± SD	**r_s_**	***p***
FS 2L	**534.11 ± 138.20**	**620.65 ± 193.87**	**0.569**	**<0.001**
**FS 1L**	**2972.24 ± 1586.65**	**2764.13 ± 1634.90**	**0.530**	**<0.001**
**FS Tan**	**1589.48 ± 773.37**	**1510.59 ± 783.48**	**0.524**	**0.001**
SS 2L	**1454.29 ± 410.34**	**1151.09 ± 264.54**	**0.465**	**0.004**
**SS 1L**	**4386.91 ± 1859.00**	**3961.14 ± 1678.25**	**0.675**	**<0.001**
**SS Tan**	**3246.75 ± 1319.72**	**2847.35 ± 1249.81**	**0.552**	**<0.001**
**v mean**	**First Round**	**Second Round**	**Spearman’s Rho**	
	Mean ± SD	Mean ± SD	**r_s_**	***p***
**FS 2L**	**2.65 ± 0.71**	**3.10 ± 0.97**	**0.596**	**<0.001**
**FS 1L**	**14.84 ± 8.04**	**13.82 ± 8.17**	**0.530**	**<0.001**
**FS Tan**	**7.69 ± 3.59**	**7.55 ± 3.92**	**0.524**	**0.001**
**SS 2L**	**7.32 ± 2.06**	**5.75 ± 1.32**	**0.465**	**0.004**
**SS 1L**	**22.12 ± 9.36**	**19.08 ± 8.39**	**0.675**	**<0.001**
**SS Tan**	**16.53 ± 6.43**	**14.24 ± 6.25**	**0.552**	**<0.001**

Two-legged stance on a firm surface (2L FS), one-legged stance on firm surface (1L FS), tandem stance on a firm surface (Tan FS), two-legged stance on a soft surface (2L SS), one-legged stance on a soft surface (1L SS), tandem stance on a soft surface (Tan SS). Not calculable (n.c.). Statistically significant results are printed in bold.

**Table 6:** Feedback results.

Questions	Very Much	Somewhat	Undecided	Not Really	Not at All	Mean ± SD	Median	Mode
Do you feel well?	17	16	2	1	0	4.36 ± 0.72	4	5
Did you enjoy the testing on the force plate?	10	19	6	1	0	4.06 ± 0.75	1	4
Was the testing on the force plate well-explained?	33	3	0	0	0	4.92 ± 0.28	0	5
Was testing on the force plate easy for you?	3	7	22	3	1	3.22 ± 0.83	3	3
	**Doctor’s visit**	**Physical education**	**Sports**	**Computer game**	**Other**			
What did the tests remind you most of?	11	11	8	3	4			

## Data Availability

The data presented in this study are available on request from the corresponding author. The data are not publicly available due to the sensitive character of pediatric clinical data.

## Supplementary Materials

The following supporting information can be downloaded at: https://www.mdpi.com/article/10.3390/diagnostics14050513/s1, Table S1, Repeated measures ANOVA for the six test positions for the instrumented BESS; Table S2, Post hoc pairwise comparison for BESS error scores in all 6 testing positions; Table S3, Post hoc pairwise comparison for EA in all 6 testing positions; Table S4, Post hoc pairwise comparison for PL in all 6 testing positions; Table S5, Post hoc pairwise comparison for VM in all 6 testing positions; Table S6, Post hoc pairwise comparison for VMml in all 6 testing positions; Table S7, Post hoc pairwise comparison for VMap in all 6 testing positions; Table S8, Comparison between VMml and VMap (paired t-test).
