# Supplementary material for: Instrumented Balance Error Scoring System in Children and Adolescents—A Cross Sectional Study
Source: Diagnostics (Basel). 2024 Feb 28;14(5):513. doi: 10.3390/diagnostics14050513 (PMC10930639; doi:10.3390/diagnostics14050513)
Supplement: Supplementary file 1 [file diagnostics-14-00513-s001.zip › diagnostics-2812517-supplementary.pdf]

Table S1

Repeated measures ANOVA for the six test positions for the instrumented BESS

Parameter	Sum of Squares	df	Mean Square	F	*p*
BESS Score	1,913.87	2.82	677.71	116.56	< .001
Ellipse Area	1,221,355.39	2.44	500,830.33	22.20	< .001
Path Length	370,641,832.00	2.35	157,528,600.00	59.07	< .001
Mean Velocity	9,276.52	2.35	3,944.11	59.18	< .001
Mean Velocity ML	312,949.97	1.96	159,521.85	25.22	< .001
Mean Velocity AP	389,125.56	2.34	166,595.37	23.04	< .001

Mean velocity in medio-lateral (Mean Velocity ML), mean velocity in anterior-posterior (Mean Velocity AP)

Table S2

Post-hoc pairwise comparison for BESS error scores in all 6 testing positions

Testing position		Mean Difference	Std. Error (SE)	*p*	95% confidence interval (CI)	
					Lower	Upper
2L FS	1L FS	-4.81	0.47	.001	-6.27	-3.35
	Tan FS	-1.54	0.27	.001	-2.39	-0.69
	2L SS	-0.84	0.17	.001	-1.36	-0.31
	1L SS	-8.38	0.29	.001	-9.29	-7.47
	Tan SS	-5.24	0.47	.001	-6.72	-3.76
1L FS	Tan FS	3.27	0.42	.001	1.95	4.59
	2L SS	3.97	0.46	.001	2.53	5.42
	1L SS	-3.57	0.40	.001	-4.81	-2.32
	Tan SS	-0.43	0.66	.999	-2.51	1.64
Tan FS	2L SS	0.70	0.29	.322	-0.22	1.62
	1L SS	-6.84	0.34	.001	-7.92	-5.76
	Tan SS	-3.70	0.50	.001	-5.26	-2.14
2L SS	1L SS	-7.54	0.33	.001	-8.57	-6.51
	Tan SS	-4.41	0.44	.001	-5.80	-3.01
1L SS	Tan SS	3.14	0.55	.001	1.39	4.88

two-legged stance on firm surface (2L FS), one-legged stance on firm surface (1L FS), tandem stance on firm surface (Tan FS), two-legged stance on soft surface (2L SS), one-legged stance on soft surface (1L SS), tandem stance on soft surface (Tan SS)

Table S3

Post-hoc pairwise comparison for EA in all 6 testing positions

Testing position		Mean Difference	Std. Error (SE)	*p*	95% confidence interval (CI)	
					Lower	Upper
2L FS	1L FS	-109.48	28.10	.006	-197.81	-21.15
	Tan FS	-19.08	5.01	.008	-34.82	-3.35
	2L SS	-19.41	1.87	.001	-25.29	-13.54
	1L SS	-213.16	27.75	.001	-300.41	-125.90
	Tan SS	-105.63	16.38	.001	-157.14	-54.13
1L FS	Tan FS	90.40	27.38	.033	4.33	176.47
	2L SS	90.07	28.48	.048	0.54	179.59
	1L SS	-103.68	32.59	.045	-206.14	-1.22
	Tan SS	3.85	36.09	.999	-109.63	117.32
Tan FS	2L SS	-0.33	5.48	.999	-17.55	16.88
	1L SS	-194.08	28.09	.001	-282.39	-105.76
	Tan SS	-86.55	18.16	.001	-143.63	-29.47
2L SS	1L SS	-193.74	27.59	.001	-280.49	-107.10
	Tan SS	-86.22	15.90	.001	-136.22	-36.22
1L SS	Tan SS	107.53	31.27	.022	9.22	205.83

two-legged stance on firm surface (2L FS), one-legged stance on firm surface (1L FS), tandem stance on firm surface (Tan FS), two-legged stance on soft surface (2L SS), one-legged stance on soft surface (1L SS), tandem stance on soft surface (Tan SS)

Table S4

Post-hoc pairwise comparison for PL in all 6 testing positions

Testing position		Mean Difference	Std. Error (SE)	*p*	95% confidence interval (CI)	
					Lower	Upper
2L FS	1L FS	-2,438.14	257.50	.001	-3,247.66	-1,628.61
	Tan FS	-1,055.37	120.97	.001	-1,435.67	-675.07
	2L SS	-920.18	64.01	.001	-1,121.42	-718.94
	1L SS	-3,852.80	306.70	.001	-4,817.03	-2,888.58
	Tan SS	-2,712.64	217.41	.001	-3,396.16	-2,029.12
1L FS	Tan FS	1,382.77	252.10	.001	590.21	2,175.33
	2L SS	1,517.95	270.00	.001	669.11	2,366.80
	1L SS	-1,414.67	243.85	.001	-2,181.29	-648.04
	Tan SS	-274.51	383.70	.999	-1,480.81	931.80
Tan FS	2L SS	135.18	129.89	.999	-273.18	543.55
	1L SS	-2,797.44	310.47	.001	-3,773.50	-1,821.38
	Tan SS	-1,657.28	261.99	.001	-2,480.95	-833.61
2L SS	1L SS	-2,932.62	301.99	.001	-3,882.04	-1,983.20
	Tan SS	-1,792.46	195.58	.001	-2,407.34	-1,177.59
1L SS	Tan SS	1,140.16	369.78	.059	-22.37	2,302.69

two-legged stance on firm surface (2L FS), one-legged stance on firm surface (1L FS), tandem stance on firm surface (Tan FS), two-legged stance on soft surface (2L SS), one-legged stance on soft surface (1L SS), tandem stance on soft surface (Tan SS)

Table S5

Post-hoc pairwise comparison for VM in all 6 testing positions

Testing position		Mean Difference	Std. Error (SE)	*p*	95% confidence interval (CI)	
					Lower	Upper
2L FS	1L FS	-12.21	1.29	.001	-16.25	-8.17
	Tan FS	-5.29	0.60	.001	-7.19	-3.40
	2L SS	-4.62	0.32	.001	-5.62	-3.62
	1L SS	-19.28	1.53	.001	-24.09	-14.47
	Tan SS	-13.58	1.09	.001	-17.00	-10.16
1L FS	Tan FS	6.91	1.26	.001	2.95	10.88
	2L SS	7.59	1.35	.001	3.35	11.83
	1L SS	-7.07	1.22	.001	-10.91	-3.24
	Tan SS	-1.37	1.92	.999	-7.40	4.66
Tan FS	2L SS	0.68	0.65	.999	-1.37	2.72
	1L SS	-13.99	1.55	.001	-18.87	-9.11
	Tan SS	-8.29	1.31	.001	-12.40	-4.17
2L SS	1L SS	-14.66	1.51	.001	-19.41	-9.92
	Tan SS	-8.96	0.98	.001	-12.05	-5.89
1L SS	Tan SS	5.70	1.85	.059	0.11	11.51

two-legged stance on firm surface (2L FS), one-legged stance on firm surface (1L FS), tandem stance on firm surface (Tan FS), two-legged stance on soft surface (2L SS), one-legged stance on soft surface (1L SS), tandem stance on soft surface (Tan SS)

Table S6

Post-hoc pairwise comparison for VMml in all 6 testing positions

Testing position		Mean Difference	Std. Error (SE)	*p*	95% confidence interval (CI)	
					Lower	Upper
2L FS	1L FS	-64.94	7.24	.001	-87.70	-42.18
	Tan FS	-26.44	2.73	.001	-35.01	-17.87
	2L SS	-45.75	15.38	.078	-94.12	2.61
	1L SS	-120.24	9.13	.001	-148.96	-91.53
	Tan SS	-68.40	5.59	.001	-85.96	-50.84
1L FS	Tan FS	38.50	6.61	.001	17.71	59.29
	2L SS	19.18	18.06	.999	-37.59	75.96
	1L SS	-55.31	8.30	.001	-81.39	-29.22
	Tan SS	-3.46	10.04	.999	-35.03	28.11
Tan FS	2L SS	-19.31	16.15	.999	-70.09	31.46
	1L SS	-93.80	8.78	.001	-121.41	-66.20
	Tan SS	-41.96	6.27	.001	-61.69	-22.24
2L SS	1L SS	-74.49	19.21	.006	-134.88	-14.10
	Tan SS	-22.65	14.55	.999	-68.39	23.09
1L SS	Tan SS	51.84	10.17	.001	19.88	83.81

two-legged stance on firm surface (2L FS), one-legged stance on firm surface (1L FS), tandem stance on firm surface (Tan FS), two-legged stance on soft surface (2L SS), one-legged stance on soft surface (1L SS), tandem stance on soft surface (Tan SS)

Table S7

Post-hoc pairwise comparison for VMap in all 6 testing positions

Testing position		Mean Difference	Std. Error (SE)	*p*	95% confidence interval (CI)	
					Lower	Upper
2L FS	1L FS	-88.04	9.88	.001	-119.11	-56.97
	Tan FS	-40.40	4.97	.001	-56.02	-24.79
	2L SS	-44.30	15.56	.109	-93.24	4.63
	1L SS	-122.61	11.05	.001	-157.35	-87.88
	Tan SS	-102.67	8.82	.001	-130.41	-74.94
1L FS	Tan FS	47.64	10.09	.001	15.91	79.37
	2L SS	43.74	19.78	.502	-18.45	105.92
	1L SS	-34.57	8.41	.003	-61.00	-8.15
	Tan SS	-14.64	15.15	.999	-62.28	33.01
Tan FS	2L SS	-3.90	17.03	.999	-57.43	49.63
	1L SS	-82.21	11.67	.001	-118.90	-45.52
	Tan SS	-62.27	10.62	.001	-95.66	-28.89
2L SS	1L SS	-78.31	20.82	.009	-143.75	-12.87
	Tan SS	-58.37	14.34	.004	-103.44	-13.30
1L SS	Tan SS	19.94	14.28	.999	-24.96	64.83

two-legged stance on firm surface (2L FS), one-legged stance on firm surface (1L FS), tandem stance on firm surface (Tan FS), two-legged stance on soft surface (2L SS), one-legged stance on soft surface (1L SS), tandem stance on soft surface (Tan SS)

Table S8

Comparison between VMml and VMap via paired t-test

Testing position	Mean difference	Std. Error (SE)	95% confidence interval (CI)		*p*
			Lower	Upper	
2L FS	2.49	0.61	1.266	3.72	.001
1L FS	-20.71	5.97	-32.72	-8.50	.001
Tan FS	-11.47	2.97	-17.49	-5.45	.001
2L SS	3.94	1.41	1.10	6.78	.008
1L SS	0.12	4.92	-9.87	10.11	.980
Tan SS	-31.78	5.57	-43.07	-20.49	.001

two-legged stance on firm surface (2L FS), one-legged stance on firm surface (1L FS), tandem stance on firm surface (Tan FS), two-legged stance on soft surface (2L SS), one-legged stance on soft surface (1L SS), tandem stance on soft surface (Tan SS)
